# Parental Stress Scale: Psychometric Properties in Parents of Preschool Children with Developmental Language Disorder

**DOI:** 10.3390/healthcare11091332

**Published:** 2023-05-05

**Authors:** Konstantinos Kotsis, Maria Boukouvala, Aspasia Serdari, Iouliani Koullourou, Vassiliki Siafaka, Thomas Hyphantis

**Affiliations:** 1 Department of Psychiatry, Faculty of Medicine, School of Health Sciences, University of Ioannina, 45110 Ioannina, Greece; 2 Department of Child and Adolescent Psychiatry, Medical School, Democritus University of Thrace, 68100 Alexandroupolis, Greece; 3 Department of Speech & Language Therapy, School of Health Sciences, University of Ioannina, 45500 Ioannina, Greece

**Keywords:** developmental language disorder, Parental Stress Scale, preschool, psychometrics

## Abstract

Parents of children with developmental disabilities experience more stress compared to those of typically developing children; therefore, measuring parental stress may help clinicians to address it. The Parental Stress Scale (PSS) is a self-rceport measure in the public domain that assesses stress related to child rearing. The present study tested the psychometric properties of the Greek version of the PSS in 204 parents (mean age: 39.4 ± 5.7, 124 mothers and 80 fathers) of kindergarten children diagnosed with Developmental Language Disorder (DLD) after a clinical assessment. Confirmatory factor analysis (CFA) was used to confirm the original four-factor structure. The results showed that the original four-factor structure (parental rewards, parental stressors, lack of control and parental satisfaction) is valid in this specific Greek population. The reliability was high (ω = 0.78) and there were weak correlations (r = −0.372, r = −0.337, r = −0.236), yet of statistical significance (*p* < 0.001), with similar psychological constructs (quality of life, emotional functioning and worries). Our data confirmed that the PSS is a reliable and valid tool to measure parental stress in parents of children with DLD. Greek clinicians (mental health professionals, speech-language pathologists) can evaluate parental stress and design early interventions targeting specific stress aspects, along with core language interventions for the children.

## 1. Introduction

Parenting is considered both a rewarding and, at the same time, demanding experience, and parental stress seems to be the rule rather than the exception [1,2]. Parental stress is the process that leads to aversive psychological reactions arising from the attempts to adapt to the demands of parenthood [3]. Parental stress levels are dynamic and depend on various factors, including the developmental state and behavior of the child [4,5,6]. For example, parental stress levels, in general, are higher among parents of children with developmental disabilities than parents of typically developing children [7]. A recent study [8] showed that parents of children with neurodevelopmental disorders reported higher levels of parenting stress compared to children of typical development. This finding in the study applied not only to Autism Spectrum Disorder (ASD) or Attention Deficit Hyperactivity Disorder (ADHD) but also to Specific Learning Disorders (SpLDs) and Language Disorders (LDs). Developmental language impairments are common in childhood, and although there is confusion about the terminology [9], it has been reported that the prevalence is estimated at around 7% in kindergarten-age children [10]. The main feature of Developmental Language Disorder (DLD) is the persistent language delay and difficulties that affect everyday communication, in the absence of any medical condition, intellectual disability or hearing impairment and with typical non-verbal abilities. Moreover, the language abilities of a child with DLD are below age expectations [9,11].

### Literature Review

Apart from impairment in verbal communication skills, children diagnosed with DLD are at risk of behavioral and emotional problems as well as impaired academic and psychosocial functioning [12,13,14,15]. Both language skills and a child’s behavior may influence parental responses [16]. Parents may feel incompetent and with a lack of confidence when their communication with their child is disturbed due to language problems. Moreover, children’s behavioral problems may evoke thoughts of incompetence and frustration due to increased difficulty to manage the behavior and finally to negative parenting [16,17]. These parental responses, in turn, influence children’s responses, reflecting the reciprocal nature of development, communication, and behavior. Since DLD can cause problems in parental–child communication, it can also cause stress in parents. Indeed, the literature suggests that mothers of children with language problems have elevated parental stress compared to mothers of children with typical language development [18]. This is a consistent finding across studies [19,20] in toddlers; parents of toddlers with language delay report higher parenting stress compared to parents of toddlers with typical development. This has been reported in very young children (18–23 months old) with poor expressive language; their mothers were more likely to worry about language development. Another study [21] found that mothers of toddlers with language delay considered the relationship with their child as more stressful compared to mothers of toddlers without language problems. In the same study, mothers of toddlers with language delay also reported higher levels of dysfunctional interactions with their child, in the parenting stress measure, compared to the same control group. Although the literature does not extend to parenting stress and LD, an Australian study [22] found that mother’s distress did not discriminate between groups (mothers of children with LD and mothers of children without LD). Furthermore, parents of preschool children with language problems may feel insecure and helpless in parenting, therefore, increasing their parental stress due to feelings of incompetence concerning the way to communicate with their child [16,17]. Moreover, it has also been reported that language development might be affected by environmental factors, such as parenting stress [23]. Even if this topic is not broadly studied, it is known that the stress parents report is associated with their children’s language problems [20]. Lastly, it is also well known that parenting stress may affect parent–child interactions, and this may impact language development [24]. Therefore, it is important for clinicians to measure parental stress levels, especially of preschool children, to be able to design the best early intervention to support parents apart from the language intervention for their children.

A brief, easy-to-administer tool, in the public domain, that measures an individual’s perception of stress, rather than the actual source of stress, is the Parenting Stress Scale (PSS) [2]. An important feature of PSS is that it was designed for use in diverse parental populations; the norms are derived from mothers and fathers of typically developing children as well as of children receiving services for emotional and/or behavioral problems. The PSS is widely used and has been translated into over 25 languages, including Greek, as well as in the general population and in clinical samples, including chronic health conditions, ADHD and ASD [25,26,27,28,29,30,31,32]. Following a principal axis factor analysis, Berry and Jones [2] found 4 factors of the 16 items (2 items were excluded) were labeled parental rewards, parental stressors, lack of control and parental satisfaction. However, other studies reported different factor structures [27,31,33]. Moreover, there are studies [27,34,35] in the literature that have altered the scale (e.g., score in a 4-point Likert scale instead of the original 5-point, drop-out items, used in grandparents). Recently, a Greek validation study [28], in mothers of healthy infants 0–12 months old, revealed two underlying factors (positive and negative aspects of parenting), confirming the validity of the scale.

Since parental stress might be different in parents of typically developing children compared to children with developmental disabilities such as DLD, this study aims to explore the four-factor structure of the PSS and analyze its psychometric properties using a sample of Greek parents having a preschool child with DLD. Using confirmatory factorial analysis (CFA), it was hypothesized that the PSS would show: (1) a four-factor solution; (2) satisfactory reliability; and (3) satisfactory convergent validity.

## 2. Materials and Methods

### 2.1. Study Design and Participants

Participants were 204 Greek-speaking parents of preschool children, who requested evaluation for language problems to a Community Child and Adolescent Mental Health Service (CAMHS), belonging to a Tertiary University Hospital. In Greece, the common pathway for children with language difficulties is to request evaluation in CAMHS, and then children enter the intervention process, either in public or private sector. All parents who requested (for their children) a speech and language evaluation were informed about the aims of the study. None of the parents (or children) were refused participation. Our CAMHS is the only public service in the region; therefore, the vast majority of the evaluations are being performed by our multidisciplinary team. All children in our sample were clinically assessed by a speech language therapist (M.B.) and then by a child and adolescent psychiatrist (K.K., I.K.) to exclude other developmental disorders, such as Autism Spectrum Disorder (ASD) or Intellectual Disability (ID). Eligibility criteria were (a) diagnosed with Developmental Language Disorder; (b) not following a speech–language intervention; (c) native Greek speakers; (d) not showing receptive language difficulties; and (e) not suffering from any medical condition or having any mental or other developmental disorder. All parents signed an informed consent form. The study was conducted according to the guidelines of the Declaration of Helsinki, and Ethical Approval was obtained from the Ethical Committee of our Institution (University Hospital of Ioannina, Reference number: 989—21 December 2020).

Mean age of parents was 39.4 ± 5.7, and there were 124 mothers and 80 fathers whose children were in kindergarten, since preschool education is obligatory in Greece. Most of the parents were married (*n* = 200, 98%) and had finished obligatory education (10 years) in Greece (*n* = 182, 89.2%). The mean Parental Stress Scale score was 30.9 ± 6.7.

### 2.2. Measures

Parental Stress Scale (PSS): PSS is an 18-item scale, which is used to identify the perceived stress resulting from being a parent [2]. Each item is scored from 1 (“strongly disagree”) to 5 (“strongly agree”) on a 5-point Likert scale. The scale consists of eight reverse-coded items, and the total possible score ranges from 18 to 90, after summing all items; higher scores indicate higher parental stress. The original factor structure consists of 4 factors, labeled: (a) parental rewards (e.g., item 1—PSS1 R “I am happy in my role as a parent”), (b) parental stressors (e.g., item 3—PSS3 “Caring for my child(ren) sometimes takes more time and energy than I have to give”), (c) lack of control (e.g., item 15—PSS15 “I feel overwhelmed by the responsibility of being a parent”), and (d) parental satisfaction (e.g., item 17—PSS17 R “I am satisfied as a parent”). Two items (2 and 4) failed to load on any of the above four factors and were removed in the original study. The Greek version [28] used for mothers of infants 0–12 months old revealed a 2-factor model with the 2 underlying factors labeled: (a) positive aspects of parenting and (b) negative aspects of parenting.

PedsQL^TM^ Family Impact module (PedsQL FIM): The 36-item PedsQL^TM^ Family Impact Module Scales [36] encompass 6 scales measuring parent self-reported functioning (physical, emotional—e.g., “I feel frustrated”, social, cognitive, communication, worry—e.g., “I worry about my child’s future”) and 2 scales measuring parent-reported family functioning (daily activities, family relationships). A 5-point response scale is utilized (0 = never a problem; 4 = always a problem). Items are reverse-scored and linearly transformed to a 0–100 scale, so that higher scores indicate better functioning (less negative impact). A total scale score, a parent Health-Related Quality of Life summary score and a family functioning summary score are generated as a result of the sum of specific items divided by the number of items answered, respectively. For the purposes (convergent validity) of the current study, we used the emotional functioning and worry subscales.

World Health Organization Quality of life Instrument, Short-Form (WHOQOl-BREF): HRQoL was assessed using the validated Greek [37] version of the WHOQoL-BREF, which assesses four domains: Physical, Psychological—e.g., “How much do you enjoy life?”, Social Relations and Environment. Each item is rated on a 5-point Likert scale and the scores are transformed on a scale from 0 to 100, where higher scores indicate better HRQOL. For the purposes (convergent validity) of the current study, we used the psychological domain.

Strengths and Difficulties Questionnaire (SDQ—Parent Version): The SDQ [38,39] is a brief behavioral screening questionnaire for completion by the parents of 4–17 year olds. SDQ asks about 25 attributes that are divided into the following 5 subscales: (a) emotional symptoms; (e.g., “Often unhappy, down-hearted or tearful”); (b) conduct problems; (e.g., often has temper tantrums or hot tempers); (c) hyperactivity/inattention; (e.g., thinks things out before acting); (d) peer relationship problems (e.g., rather solitary, tends to play alone); and (e) prosocial behavior. Responses to each of the 25 items consisted of 3 options: not true, somewhat true or certainly true. The first four subscales added together generate a total difficulty score, which was used in the present study

### 2.3. Statistical Analysis

The Statistical Package for Social Sciences v28 for MacOS, Omega macro for SPSS and JASP software were used [40,41,42]. McDonald’s omega (ω) and Cronbach’s alpha (α) were calculated to analyze the internal consistency of the PSS. Confirmatory factor analysis (CFA) was performed to verify the factorial structure of the questionnaire in our sample of parents of children with DLD. In this analysis, we tested the 4-factor model proposed in the original study of Berry and Jones [2]. The goodness of fit was assessed using the following indices and corresponding thresholds: (a) the ratio of the chi-square statistic to the respective degrees of freedom (χ^2^/df) ≤ 2, which assesses overall fit and the discrepancy between the sample and fitted covariance matrices, (b) the values of the parsimony-adjusted index Root Mean Square Error of Approximation (RMSEA) ≤ 0.06, (c) the Normed Fit Index (NFI) ≥ 0.90, which indicates that the model of interest improves the fit, (d) the Comparative Fit Index (CFI) ≥ 0.90 and (e) Tucker–Lewis index (TLI) ≥ 0.90. Convergent validity was assessed by calculating the correlation of scores between the PSS-18 and the parental emotional functioning and parental worry dimensions of PedsQL^TM^ Family Impact and psychological HRQoL of WHOQoL-BREF, using the Spearman’s correlation coefficient.

Network analysis, conducted in JASP software Version 0.17.1, was used to study interaction between the different parental stress items. A network is represented by nodes (e.g., items) and the relationships between them, called edges (positive blue and negative red associations). Thicker and higher-color-density edges indicate a stronger connection among nodes [43]. Highly connected nodes usually cluster and these are equivalent to latent factors [44].

## 3. Results

### 3.1. Reliability

The reliability of the PSS was examined by considering the unstandardized McDonald’s ω, and Cronbach’s α revealed a value of 0.78 and 0.77, respectively, which is considered high. The total scale without item PSS 09 resulted in a slight increase in reliability estimates (ω = 0.79 and α = 0.78). Item total correlation showed positive relationships (data not shown), with values over 0.3, except items PSS_2, PSS_4 (which were omitted in most previous studies), PSS_7 and PSS_9, which showed values of 0.2.

### 3.2. Factor Analysis

**Figure 1:**
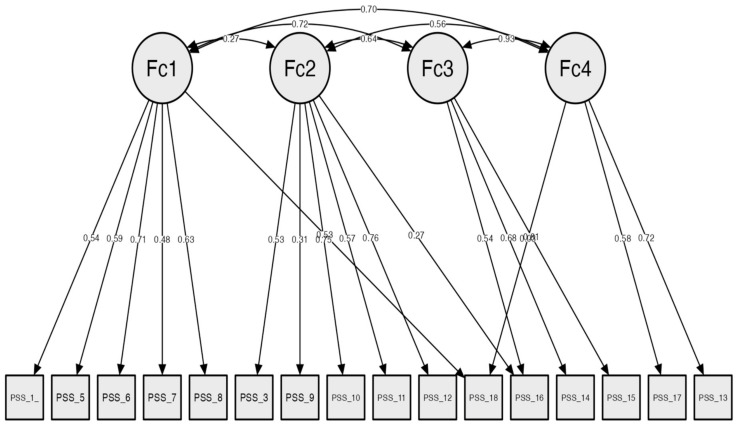
Confirmatory factor analysis for Parental Stress Scale, original 4-factor model. Fc1 = parental rewards; Fc2 = parental stressors; Fc3 = lack of control; Fc4 = parental satisfaction.

CFA was applied, and the goodness-of-fit indices (Table 1) revealed factorial validity for the four factors, as suggested in previous research (χ^2^/df = 1.27, *p* = 0.04; RMSEA = 0.04; NFI = 0.94; CFI = 0.98; TLI = 0.98). The model plot and item factorial weights for each factor are presented in Figure 1. The original factors were called parental rewards, parental Stressors, lack of control and parental satisfaction.

### 3.3. Convergent Validity

Convergent validity was considered by examining the correlations between the PSS and parental emotional functioning, parental worry and psychological HRQoL. The results (Table 2) showed significant correlations in the expected directions. PSS total score was negatively correlated with the Psychological HRQoL (r = −0.372, *p* < 0.001), the Family Impact emotional functioning subscale (r = −0.337, *p* < 0.001), and less strongly with the Family Impact Worry subscale (r = −0.236, *p* < 0.001).

### 3.4. Network Analysis

**Figure 2:**
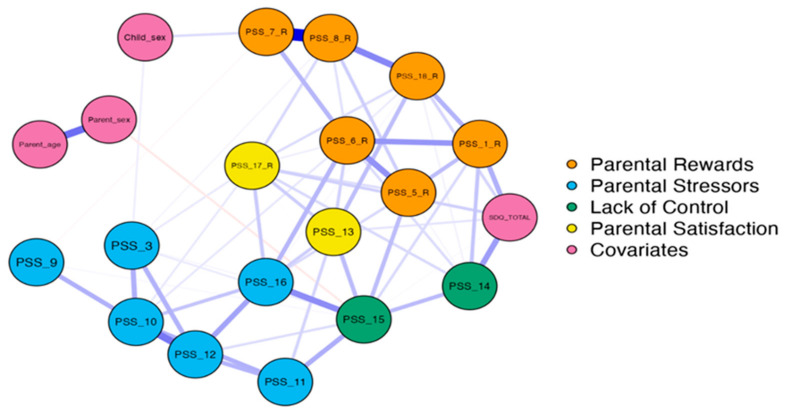
Network analysis of 16 items on Parental Stress Scale and covariates. Blue lines represent positive associations and red lines negative. Edge brightness and thickness reflect the strength of association.

Network analyses were performed to explore how the 16 PSS (items PSS_02 and PSS_04 were omitted) items connect and interact with each other as well as with four covariates (parental sex, parental age, child sex, SDQ total score). Figure 2 shows the network among the 16 items (blue, green, yellow, orange) and the covariates (pink). Supplementary Table S1 shows the correlation matrix among the items. Item loading on different factors clustered together, supporting that this represents separate factors. As shown, the nodes in the vast majority are positively connected, with several strong connections, e.g., PSS_7 (my child[ren] is an important source of affection for me) and PSS_8 (having child[ren] gives me a more certain and optimistic view for the future) or PSS_10 (having child[ren] leaves little time and flexibility in my life) and PSS_12 (it is difficult to balance different responsibilities because of my child[ren]), indicating that these statements tend to co-exist. A weak negative association was noted between parental sex and PSS_15 (I feel overwhelmed by the responsibility of being a parent), indicating that mothers tend to feel more overwhelmed compared to fathers. Emotional and behavioural difficulties (SDQ total score) were positively associated, though not strongly, with items PSS_1 (I am happy in my role as a parent—reversed) and PSS_14 (if I had it to do over again, I might decide not to have children), indicating that parents who reported more problems for their child tended to report more difficulties for themselves as parents. Demographics were peripheral to the network and did not associate strongly with any specific node in the network. However, there was a weak negative association of parents’ sex with item PSS_15 and weak positive associations of child’s sex with items PSS_3 and PSS_7_R.

**Figure 3:**
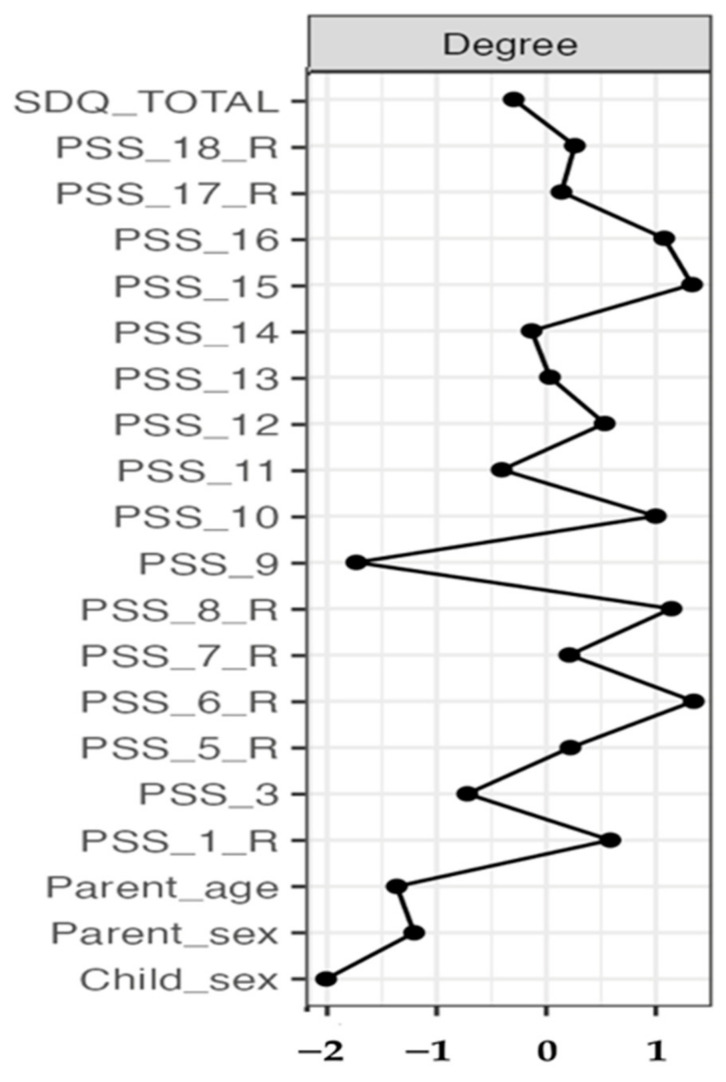
Node degree centrality of network analysis.

Figure 3 shows the degree strength centrality (Figure 3), which represents the number of edges connected to the node; the higher the score, the more likely the node is to receive and affect other nodes in the network. The nodes with the highest degree centrality were PSS_15 (I feel overwhelmed by the responsibility of being a parent) and PSS_6 (I enjoy spending time with my child[ren]—reversed).

## 4. Discussion

The aim of the current study was to assess whether the PSS could be used for parents of preschool children with DLD by exploring its validity and reliability. The results show that parental stress in this population is better conceptualized as the four original factors. Moreover, reliability was found to be high, and convergent validity was supported by the correlation, in the expected direction, with similar parental psychological constructs.

Our findings replicate the factor structure of the PSS, confirming the four subscales, which can be conceptualized as positive (parental rewards and parental satisfaction subscales) and negative (parental stressors and lack of control) experiences. This is in line with previous research where PSS analysis revealed the same four-factor structure in a sample of parents of children with chronic health conditions and children 3–10 years old in a population sample [29,31]. Specifically, Zelman et al. [29] replicated the original four-factor structure in children with newly diagnosed asthma, diabetes, epilepsy, food allergy and juvenile arthritis, concluding that the PSS is a valid and reliable tool for these parents. Some studies in the literature [26,27,28] identified a two-factor solution as the best model fit; however, Pontoppidan et al. [26] suggested that the four factors may represent subdivisions of two factors, called parental stressors and parental satisfaction. The same hypothesis was stated in the original study, where Berry and Jones [2] supported the dichotomy (positive and negative) of the parenting experiences.

The reliability of the scale was found to be high, in line with previous studies, both in the general population [27,28,31] and in parents in diverse clinical samples [30,32]. Convergent validity of the PSS was also demonstrated in this study. As hypothesized, parental stress was correlated with similar psychological constructs, such as emotional functioning, psychological quality of life and the worries reported by parents concerning the family impact of the DLD. These relationships point out that parental stress is characterized by anxiety, sadness, anger, frustration and feeling helpless or hopeless (emotional functioning). Parental stress also may affect the psychological quality of life, raising positive and negative feelings and impacting parental self-esteem (psychological quality of life). Worries about a child’s treatments and side effects, about others’ reactions to a child’s condition and about a child’s future correlated weakly with parental stress in our sample. Moreover, the other constructs also associated weakly with parental stress. Weak correlation may be explained by the fact that DLD is not considered an illness that needs treatment but a diversity that needs intervention. However, in general, associations in our sample are in line with numerous studies reporting a correlation between parental stress and psychological distress, although research refers mainly to parents of children with medical conditions [29,45,46,47].

Findings from the network analysis added support to the four-factor structure, as all items loaded on each different factor were clustered. Moreover, the items on the PSS were positively correlated, although with different strength. Concerning the first factor (parental rewards), connections were generally strong with item PSS_7 (my child[ren] is an important source of affection for me) and PSS_8 (having child[ren] gives me a more certain and optimistic view for the future), showing a stronger connection. Moreover, strong connections were revealed between PSS_8 (having child[ren] gives me a more certain and optimistic view for the future) and PSS_18 (I find my child[ren] enjoyable) as well as PSS_1 (I am happy in my role as a parent) with PSS_5 (I feel close to my child[ren]) and PSS_6 (I enjoy spending time with my child[ren]). These items (loaded on the same factor—parental rewards) tended to co-occur, indicating that when parents enjoy their life with their children, they are happier and more optimistic. Similarly, within the parental stressors factor, there were strong connections, such as PSS_10 (having child[ren] leaves little time and flexibility in my life) with PSS_12 (it is difficult to balance different responsibilities because of my child[ren]). This connection seems rather logical since, for parents with less time, they may easily find themselves struggling between various responsibilities. Items from the lack of control factor and parental satisfaction were also connected but less strongly. However, the feeling of overwhelming in parents tended to co-occur with the feeling of having few choices and little control over their lives (note that, although PSS_16 is a different color, it also loads on the factor lack of control, which is green). The only negative connection apparent in the network was between parents’ sex and PSS_15 (I feel overwhelmed by the responsibility of being a parent), implying that mothers tend to feel that it is harder for them to deal with the responsibility of being a parent compared to fathers. However, the association was very weak. This is in line with Greek culture, in which, usually, the father handles all financial matters and the mother takes care of the children [48]. Parent reports concerning emotional and behavioral difficulties in their children were positively associated with items PSS_1 (I am happy in my role as a parent—reversed) and PSS_14 (if I had it to do over again, I might decide not to have children). These connections are consistent with the broader literature, showing that parents of children with mental health or developmental problems report a lower quality of life compared with parents of healthy children [49]. Finally, the items/nodes with the highest strength were PSS_15 (I feel overwhelmed by the responsibility of being a parent) and PSS_6 (I enjoy spending time with my child[ren]—reversed). Therefore, these, central to the network, items play a major role in parental stress and may represent important targets when it comes to identifying and helping parents of children with DLD who struggle with stress in their parental role.

### Strengths and Limitations

The strengths of our study are two-fold: first, the specific diagnostic group and, second, the age spectrum of the children. Focusing on the preschool period and helping parents concerning their stress may be beneficial for their children too, to facilitate communication. Moreover, the sample that consisted of parents of children with DLD is important, since most studies explore the validity of the PSS in the general population. Exploring that in a specific population may provide clinicians with valuable information.

However, our results should be interpreted in the light of some limitations. First, there was no control group (either with no communication disorders or with other simple developmental disorders) to compare parental stress; therefore, we are not aware if our sample presented with higher-than-average levels of parental stress. However, there are no established cut-off scores for the PSS [25,26,27], and this could be an area of future research. Furthermore, the lack of a control group limits our ability to explore if the PSS could differentiate between parents of children with and without DLD. Moreover, we assessed the convergent validity by exploring the correlation with emotional functioning and psychological HRQoL, which are similar constructs, yet the correlation with an instrument measuring parental stress would be more appropriate. Finally, the generalizability of our results is quite limited due to the specific population and language.

## 5. Conclusions and Clinical Implications

Our study adds to the existing literature on the psychometric properties of the PSS, expanding its use in a specific population. We found that the PSS is a reliable and valid tool for parents of children with DLD and consists of four subscales, as proposed by Berry and Jones [2]. The PSS can be used to assess the level of parental stress and any lack of parental satisfaction and determine their balance, experienced by parents of preschool children with DLD.

Mental health professionals involved in the care of children with DLD can use the PSS to evaluate parental stress. The evaluation could inform specific family-centered psychoeducational interventions in parents of children with DLD. Professionals using the PSS might be in a position to identify specific positive and negative parenting experiences and intervene to help parents who struggle in the child rearing and concurrently reinforce aspects of parental satisfaction and rewards. Moreover, as some items in the PSS seem more central than others in the formation of parental stress, these may represent the main targets of the interventions. By improving satisfaction and reducing stress, parents may feel less overwhelmed and happier, and this may facilitate communication between parent and child.

## Figures and Tables

**Table 1:** Assessment of model fit.

Measure	Threshold	4-Factor Model
χ^2^/df	≤2.0	1.27
RMSEA	≤0.06	0.04
NFI	≥0.90	0.94
CFI	≥0.90	0.98
TLI	≥0.90	0.98

χ^2^ = chi-square; df = degrees of freedom; RMSEA = Root Mean Square Error of Approximation; NFI = Normed Fit Index, CFI = Comparative Fit Index; TLI = Tucker–Lewis index.

**Table 2:** Correlations between PSS total score, Psychological HRQoL domain of WHOQoL-BREF, Emotional Functioning and Worry subscales of PedsQL^TM^ Family Impact.

	PSS Total Score	WHOQoL Psychological	FI Emotional Functioning	FI Worry
PSS total score	-			
WHOQoL Psychological	−0.372 ***	-		
FI Emotional Functioning	−0.337 ***	0.499 ***	-	
FI Worry	−0.236 **	0.261 ***	0.471 ***	-

*** Correlation is significant at the 0.001 level, ** *p* < 0.01

## Data Availability

Data are available from the authors upon request.

## Supplementary Materials

The following supporting information can be downloaded at: https://www.mdpi.com/article/10.3390/healthcare11091332/s1, Table S1: Correlation matrix among PSS items, SDQ total score and demographics.
