# Supplementary material for: Parental Stress Scale: Psychometric Properties in Parents of Preschool Children with Developmental Language Disorder
Source: Healthcare (Basel). 2023 May 5;11(9):1332. doi: 10.3390/healthcare11091332 (PMC10178680; doi:10.3390/healthcare11091332)
Supplement: Supplementary file 1 [file healthcare-11-01332-s001.zip › healthcare-2343893-supplementary.pdf]

Table S1: Correlation matrix among PSS items, SDQ total score and demographics.

	Network																			
**Variable**	**Child_sex**	**Parent_sex**	**Parent_age**	**SDQ_tot**	**PSS_1_R**	**PSS_3**	**PSS_5_R**	**PSS_6_R**	**PSS_7_R**	**PSS_8_R**	**PSS_9**	**PSS_10**	**PSS_11**	**PSS_12**	**PSS_13**	**PSS_14**	**PSS_15**	**PSS_16**	**PSS_17_R**	**PSS_18_R**
Child_sex						0.030			0.064											
Parent_sex			0.258														-0.040			
Parent_age		0.258																		
SDQ_total					0.142										0.048	0.174	0.006		0.084	0.074
PSS_1_R				0.142			0.118	0.188								0.103	0.066			0.134
PSS_3	0.030									0.017		0.144		0.137			0.037	0.015	0.039	
PSS_5_R					0.118			0.194		0.087						0.016	0.117	0.067	0.060	
PSS_6_R					0.188		0.194		0.129	0.052		0.042			0.079	0.063	0.003	0.129	0.031	0.034
PSS_7_R	0.064							0.129		0.452	-0.010									
PSS_8_R						0.017	0.087	0.052	0.452										0.068	0.214
PSS_9									-0.010			0.142					0.014			
PSS_10						0.144		0.042			0.142		0.142	0.241				0.104	0.040	
PSS_11												0.142		0.135	0.076		0.147			
PSS_12						0.137						0.241	0.135				0.061	0.163		
PSS_13				0.048				0.079					0.076				0.118	0.087	0.088	0.115
PSS_14				0.174	0.103		0.016	0.063									0.122		0.068	0.024
PSS_15		-0.040		0.006	0.066	0.037	0.117	0.003			0.014		0.147	0.061	0.118	0.122		0.205	0.003	
PSS_16						0.015	0.067	0.129				0.104		0.163	0.087		0.205		0.093	0.011
PSS_17_R				0.084		0.039	0.060	0.031		0.068		0.040			0.088	0.068	0.003	0.093		0.063
PSS_18_R				0.074	0.134			0.034		0.214					0.115	0.024		0.011	0.063	
